# COVID-19 and CKD: Employment, Food Security and Healthcare in El Salvador

**DOI:** 10.5334/aogh.3820

**Published:** 2022-11-09

**Authors:** Jessica H. Leibler, Sinead A. Keogh, Emmanuel Jarquín, Ramon Garcia-Trabanino, Juan José Amador Velázquez, Damaris Lopez Pilarte, Marvin Beltran, Iris S. Delgado, Zoe E. Petropoulos, David J. Friedman, Daniel R. Brooks, Madeleine K. Scammell

**Affiliations:** 1 Department of Environmental Health, Boston University School of Public Health, Boston, MA, USA; 2 Agency for Agricultural Health and Development (AGDYSA), San Salvador, El Salvador, SV; 3 Centro de Hemodiálisis, San Salvador, El Salvador, SV; 4 Department of Epidemiology, Boston University School of Public Health, Boston, MA, USA; 5 Beth Israel Deaconess Medical Center, Division of Nephrology, Boston, MA, USA

**Keywords:** COVID-19, chronic kidney disease, Mesoamerican nephropathy, food insecurity, Central America

## Abstract

**Background:**

In Central America, the COVID-19 pandemic coexists with a devastating epidemic of chronic kidney disease of unknown origin. The consequences of these overlapping health crises remain largely unknown.

**Methods:**

We assessed vulnerability to and impact of the first wave of COVID-19 on participants in a cohort study of chronic kidney disease (CKD) in El Salvador (n = 229). Participants were contacted by phone during August and September 2020. We queried changes to employment, healthcare access, household income and food security due to the pandemic (from March 2020 until the time of the interview) and COVID-19-associated symptoms during that time.

**Findings:**

We reached 94% of the cohort (n = 215). Nearly 40% of participants reported an unexpected change in employment or work activities and 8.8% reported new unemployment due to the pandemic. Participants with CKD (n = 27) had higher odds of reporting new income insecurity, food insecurity, and reductions in medical care access due to the pandemic. COVID-19-associated symptoms (an approximation of disease) were reported in 7.0% (n = 15). Participants with CKD were more likely to report COVID-19-associated symptoms compared to those without CKD, although these differences were not statistically significant.

**Conclusions:**

Overall, participants with CKD suffered greater economic consequences as a result of the pandemic and may have experienced higher incidence of COVID-19 disease, although laboratory diagnostics would be required to draw this conclusion. Longitudinal analyses are required to comprehensively evaluate the implications of the pandemic for individuals with CKD in Central America.

## Introduction

As of March 2022, COVID-19 has caused over 450 million reported cases and more than six million deaths worldwide [1]. While staggering, these statistics likely reflect underreporting of the pandemic’s effects on mortality and morbidity in many regions around the world. Limited testing, incomplete record keeping, and reduced access to healthcare contribute to imprecise estimates of the burden of COVID-19 in low- and middle-income countries in particular. Existing socioeconomic stressors exacerbated the effects of the pandemic on health, development, and wellbeing around the world, with worse outcomes among individuals experiencing poverty [2]. These challenges are particularly acute in Central America [3].

As of December 2021, COVID-19 was a leading cause of death in four Mesoamerican nations (Costa Rica, Belize, Panama and Mexico), surpassing heart disease, cancers and other chronic diseases [4]. Social and economic conditions endemic in many regions of Central America, including widespread poverty, poor housing, social unrest, underfunded health systems and government instability, created an optimal environment for rapid and deadly transmission of the virus. Concurrent epidemics of endemic infectious diseases, notably dengue, may also increase vulnerability and complicate recovery [5]. As such, the comprehensive implications of the pandemic in Central America remain unknown and are likely specifically detrimental to the health and wellbeing of people living in poverty.

In El Salvador, the government’s rapid response to the pandemic and stringent lockdown in the spring of 2020 was one of the strictest in the world. On March 11, 2020, government leaders instituted broad shutdowns of all public services to mitigate transmission of SARS-CoV-2 within the country. These quarantine measures remained in place for nearly three months. As of October 2022, the country of approximately 6.5 million has reported more than 202,000 cases and more than 4,230 deaths [6]. For comparison, El Salvador is similar in size and population to Massachusetts in the United States, which has experienced over 2.07 million reported COVID-19 cases and 21,840 deaths [7].

In El Salvador, the COVID-19 pandemic exists concurrently with a devastating epidemic of chronic kidney disease primarily affecting young working-age men living along the Pacific coast. Referred to globally as chronic kidney disease of unknown origin (CKDu) or Mesoamerican Nephropathy (MeN), it is not preceded by traditional risk factors including diabetes or hypertension [8]. CKD (or CKDu/MeN) is a leading cause of death among working-age men in El Salvador, and despite more than a decade of research, the etiology of the disease remains unclear [9,10]. At the start of the COVID-19 pandemic in March 2020, we were in the midst of a longitudinal study of male workers in El Salvador (the Mesoamerican Nephropathy Occupational Study; MANOS) to examine risk factors for kidney disease and renal injury [11]. The objectives of MANOS were to evaluate the effects of heat exposure, pesticides, and heavy metals on renal function over time within this population at high risk for CKD [12]. In 2018, we enrolled 279 men currently working in agricultural and non-agricultural industries including sugarcane (40%), corn production (40%), and road construction (20%). Participants were monitored for three consecutive workdays. Urine and serum samples were collected prior to and after the workday and kidney function was determined based on the estimated glomerular filtration rate (eGFR). Participants’ ages at baseline ranged from 18-45 years (mean: 29 years). All workers were followed every six months, at which point serum and urine samples were collected and assessed for kidney function. As of March 2020, four complete rounds of MANOS had been completed. Of the 279 MANOS participants recruited at baseline, 229 (82%) were actively participating in the study at this time.

At each round of follow-up, we shared clinical laboratory results of the prior round with each individual. Participants with eGFR < 60 mL/min/1.73 m^2^ were referred to health care and counseled by study staff. We used the KDIGO diagnostic criteria for CKD to identify individuals with kidney disease within MANOS. The diagnosis is neutral regarding etiology or cause. For this reason, we use the term “CKD” and not “CKDu” in this manuscript. Follow-up visits took place at six-month intervals from January 2018 to November 2019 (prior to the pandemic).

While CKD was identified early on as a risk factor for COVID-19 infection and severity in high-income countries [13,14], there is limited work to date regarding CKD and incidence or severity of COVID-19 in low- or middle-income countries. At baseline enrollment for the MANOS study (2018), 10.1% of the El Salvador cohort experienced CKD in age-adjusted estimates [15]. MANOS participants were predominantly considered ‘essential’ workers and live in rural communities. We were concerned about their continued employment during the pandemic and risk of SARS-CoV-2 exposure on the job.

To maintain contact and assess the vulnerability of our study participants during the first wave of COVID-19, we administered questionnaires via phone with MANOS participants in El Salvador in August and September 2020. We hypothesized that MANOS participants were impacted broadly by the pandemic, through changes in employment, healthcare access, and income or food insecurity. We also hypothesized that participants with CKD, due to loss of healthcare access, and those working in non-agricultural industries, due to loss of employment, would experience worse economic and occupational impacts of the pandemic compared to others. We considered associations between self-reported symptoms of COVID-19 and important lifestyle and quality of life changes with our most recent kidney health data, specifically comparing the experiences of our participants with CKD to those without CKD.

## Methods

### Phone interviews

Salvadoran MANOS investigators initiated phone contact with study participants actively enrolled at this time in the MANOS cohort in El Salvador (n = 229) during August and September of 2020. Interviews were conducted in Spanish by Salvadoran MANOS team members who were not blinded to participant CKD status, as they were engaged in the past in reporting eGFR data back to participants. All study protocols were approved by the Institutional Review Board at Boston University Medical Campus (#H-35819) and the Salvadoran National Ethics Committee for Health Research (*Comité Nacional de Ética de las Investigaciones en Salud*). All methods were carried out in accordance with relevant guidelines and regulations and all participants provided informed consent.

### Questionnaire

To assess participants’ experiences during the early months of the pandemic, we asked participants whether they experienced a list of 12 COVID-19-associated symptoms and whether they had obtained a COVID-19 test or diagnosis from March 2020 to the time of the interview (August or September 2020). Symptom questionnaires were adapted from those collected in the PhenX COVID-19 Protocol library to reflect those symptoms commonly experienced by diagnosed COVID-19 patients [16]. We asked participants to discuss whether their employment had changed specifically due to the COVID-19 pandemic from March 2020 until the time of the interview. We included questions regarding social distancing and PPE use to reduce risk of disease transmission while at work; hand hygiene and availability of sanitizer; and changes in food access, household income and access to medical care since March 2020.

### Data analysis

Changes to employment, use of PPE and physical distancing, food insecurity, income insufficiency and access to healthcare were evaluated descriptively in sum and by industry. Participants who reported three or more concurrent COVID-19-associated symptoms were designated as a suspected cases. We evaluated incidence of three or more concurrent self-reported COVID-19-associated symptoms during the study period as an approximation of disease incidence. We compared approximated disease incidence between participants with and without CKD. Per our existing protocols, kidney function was determined using serum creatinine to estimate glomerular filtration rate (eGFR) using the serum creatinine-based CKD-EPI equation [17]. CKD was defined using the standard KDIGO definition as eGFR values of <60 mL/min/1.73 m^2^ at two time points separated by at least three months [18]. We considered a participant to have CKD if they had two eGFR values <60 mL/min/1.73 m^2^ in two of the first four rounds of our study follow-up, each separated by six months, from 2018–2020. We used age-adjusted logistic regression (>40 years, Y/N) to compare odds of new food insecurity, income insufficiency and reduced access to medical care, comparing participants with CKD to those without CKD. Logistic regression was also used to explore the association between three or more symptoms associated with COVID-19 infection and current agricultural work (Y/N) and concurrent CKD (Y/N), after adjusting for age. All analyses were conducted in Stata IC/16.1.

## Results

Our team successfully reached 215 participants with the COVID-19 phone questionnaire (response rate: 93.9%). Median age was 30 years (range 20–47 years; SD: 7.8) and 16.7% of participants (n = 36) were 40 years or older. As earlier noted, all participants were male. Among those in the COVID-19 study, 27 participants (12.6%) were identified as having CKD on the basis of eGFR measurements from samples collected prior to the pandemic.

### Employment status

One hundred thirty-six participants (62.3%) were actively employed in agricultural industries at the time of the phone interview, including 35 (16.3%) in sugarcane, 89 (41.4%) in corn production and eight (3.7%) in mostly small-scale subsistence farming (Table 1). The majority of participants employed in non-agricultural industries worked in road construction (n = 38; 17.7%) with the remainder in miscellaneous jobs (n = 26; 12.1%). At the time of the COVID-19 study, 19 participants (8.8%) reported unemployment (Table 1).

**Table 1:** Occupation of MANOS COVID-19 study participants in El Salvador^1^.


INDUSTRY OF EMPLOYMENT	MANOS BASELINE (2018; n = 279) n (%)	COVID-19 STUDY (2020; n = 215) n (%)	CHANGE DUE TO THE PANDEMIC^2^ (MARCH-SEPTEMBER 2020) n (%)^3^

Sugarcane	111 (39.8)	35 (16.3)	10 (28.6)

Corn production	110 (39.4)	89 (41.4)	19 (21.4)

Road construction	58 (20.8)	38 (17.7)	14 (36.8)

Other – non-agriculture	0	26 (12.1)	15 (65.2)

Other – agriculture	0	8 (3.7)	4 (50.0)

Unemployed	0	19 (8.8)	19 (100.0)


^1^ Median age is 30 years (range: 20–47 years). ^2^ Reflected unexpected and unplanned changes to regular employment. ^3^ Percentage reflects workers reporting employment change/industry at time of study.

### Pandemic-related work changes

Over 38% of MANOS participants (n = 81) reported a change in their job due to the pandemic (Table 1). The largest shifts were observed among participants who were working in small-scale agriculture or non-agricultural industries at the time of interview. All participants who reported that they were unemployed indicated that they had been employed prior to the pandemic (n = 19), with the recent unemployment most common among workers previously employed in road construction.

### Income insecurity

Twelve participants (5.6%) reported severe reductions to household income due to the pandemic (which we termed new income insecurity) to the extent that they were newly unable to afford basic necessities (Table 2). Eight of these 12 individuals were employed at the time of interview. Workers in corn production and those unemployed were most likely to report new income insecurity (Table 2). Participants with CKD experienced more than ten times the odds of reporting new income insecurity (OR: 10.8; 95%CI: 2.8, 41.9) compared to those without CKD (Table 2) in models adjusted for age.

**Table 2:** Food insecurity, income insecurity and reduced access to medical care due to the COVID-19 pandemic experienced by MANOS participants in El Salvador, March-September 2020 (n = 215).


		BY CKD STATUS^1^			BY INDUSTRY OF EMPLOYMENT^2^			
	
	TOTAL (n = 215) n (%)	PARTICIPANTS WITH CKD (n = 27)n (%)^1^	PARTICIPANTS WITHOUT CKD (n = 188)n (%)	ODDS RATIO (95%CI)^3^	SUGARCANE (n = 35)n (%)	CORN (n = 89) n (%)	ROAD CONSTRUCTION (n = 38)n (%)	UNEMPLOYED (n = 19) n (%)

New food insecurity^4^	31 (14.4)	8 (29.6)	23 (12.2)	5.2 (1.8, 15.2)	4 (11.4)	15 (16.9)	4 (10.5)	6 (31.6)

New income insecurity	12 (5.6)	5 (18.5)	7 (3.7)	10.8 (2.8, 41.9)	2 (5.7)	4 (4.5)	2 (5.3)	4 (21.1)

New moderate or severe reductions in access to medical care	67 (31.2)	12 (44.4)	55 (29.3)	2.2 (0.89, 5.3)	8 (22.9)	28 (31.5)	14 (36.8)	9 (47.4)


^1^ CKD defined as eGFR measurements of <60 mL/min/1.73 m^2^ at 2+ study visits with a six-month gap from 2018–2020. ^2^ Counts for individuals reporting other industries of employment not presented due to small counts. ^3^ OR reflecting magnitude of effect comparing participants with CKD to those without CKD. Results generated from age-adjusted logistic regression. ^4^ Differences in incidence of food insecurity, income insecurity and reduced access to medical care did not differ by industry in Fisher’s Exact Tests (p > 0.05).

### Food insecurity

Thirty-one participants (14.4%) indicated that due to the pandemic their households experienced either occasional or frequent food insecurity, defined as occasionally or frequently having not enough food for household members (Table 2). Of those reporting new food insecurity, the majority were employed at the time of the interview (n = 25; 80.7%) (Table 2). Corn production workers accounted for the majority of participants reporting food insecurity, although nearly 1/3 of unemployed participants also reported shortages of food. Participants with CKD had more than five times the odds of reporting food insecurity compared to those without CKD (OR: 5.2; 95%CI: 1.8, 15.2).

### Reductions in access to medical care

Sixty-seven individuals (31.2%) indicated that they had newly experienced moderate or severe reductions in access to medical care due to the pandemic (Table 2). Of these participants, 44 (65.7%) indicated that these reductions in medical care access resulted in moderate impact on their health while 23 (34.8%) indicated a severe negative impact on their health from limited access to medical care. Individuals who made these reports were primarily unemployed, or working in corn production or road construction. Participants with CKD had more than two times the odds of reporting new moderate or severe reductions to their medical care compared to those without CKD in an age-adjusted model (OR: 2.2; 95%CI: 0.89, 5.3) (Table 2).

## COVID-19 symptoms and testing

Eighteen participants (8.4%) reported three or more COVID-19-associated symptoms (Table 3). COVID-19 testing was reported by 15 participants, all of whom reported testing negative. This tested group included three participants who reported ≥3 COVID-19-associated symptoms who were subsequently considered negative in our analyses. Excluding these individuals, 15 participants (incidence: 7.0%; 95%CI: 4.0, 11.2) had both 3+ COVID-19-associated symptoms and did not report a negative test and were subsequently considered potential COVID-19 cases (Table 3). Incidence of ≥3 COVID-19-associated symptoms among participants without CKD was 6.4 % (95%CI: 3.6, 10.9) and 11.1% (95%CI: 3.4, 30.6) among participants with CKD (Table 3). Among the participants with ≥3 COVID-19-associated symptoms, 93.3% reported fever, 73.3% reported cough, 73.3% reported sore throat, 53.3% reported loss of smell, 40.0% reported muscle aches and 33.3% reported fatigue. We observed highest incidence of COVID-19 symptoms in sugarcane workers (n = 5; 14.3%).

**Table 3:** Characteristics and risk factors for ≥3 COVID-19-associated symptoms among MANOS participants in El Salvador, March-September 2020 (n = 215).


	CUMULATIVE INCIDENCE (95% CI)

≥3 COVID-19 associated symptoms	7.0 (4.0, 11.2)

Without CKD	6.4 (3.6, 10.9)

With CKD	11.1 (3.4, 30.6)

Risk factors for ≥3 COVID-19 associated symptoms	**Odds Ratio (95% CI)** ^1^

Agricultural employment at time of interview	0.77 (0.26, 2.3)

CKD	1.2 (0.27, 5.5)


^1^ Odds ratios generated from age-adjusted logistic regression.

### Risk factors for ≥3 COVID-19 associated symptoms

In age-adjusted logistic regression, agricultural work was associated with a 23% reduction in odds of COVID-19-associated symptoms compared to non-agricultural work (OR: 0.77; 95%CI: 0.26, 2.3) (Table 3). CKD status was associated with a 21% increase in the odds of COVID-19-associated symptoms (OR: 1.2; 95%CI: 0.27, 5.5) in an age-adjusted model. These associations were not statistically significant at the p < 0.05 level. Mean number of reported COVID-19-associated symptoms did not differ by CKD status.

## Discussion

Our findings indicate that working-aged men in El Salvador experienced notable instability in employment, new income and food security, and reduced access to medical care during the early months of the COVID-19 pandemic. Participants with CKD appeared to suffer greater economic hardships associated with pandemic-related disruptions and may have experienced incidence of COVID-19 disease, although laboratory diagnostics would be required to draw this conclusion.

Approximately 40% of study participants reported a change to their work due to the pandemic, with nearly 9% indicating new unemployment due to the pandemic. These findings are suggestive of widespread employment instability in the region and may be particularly acute for workers who are employed through informal arrangements or seasonal contracts. While all MANOS participants were employed in a defined set of industries at baseline enrollment in 2018 (sugarcane, corn production, and road construction), reports of participants changing work due to the pandemic to be employed across a larger variety of industries and jobs indicates that some study participants sought non-traditional, perhaps temporary, work during this period. The long-term implications of these findings are important areas for future study, both into the later months of the pandemic and in the post-pandemic economy. Despite these employment shifts, we note that more than 90% of MANOS participants were actively employed when contacted for this study, reinforcing the ‘essential’ nature of their work and that many working age men in the country did continue to work in spite of the pandemic lockdown.

Despite uncertainty around our estimates of COVID-19 disease, incidence of COVID-19-associated symptoms in the cohort in El Salvador from March through September 2020 (7.0%) appears high in comparison to the country as a whole. For reference, as of December 2021, the total positive case count represents 1.2% of the country’s population. Our findings are clearly limited by our symptoms-based assessment, which provides an imperfect estimation of disease incidence compared to laboratory-based diagnostics (serology, antigen tests, or molecular/PCR tests) which were in limited supply in the region at the time. Our use of a symptoms-based estimation of infection is consistent with other efforts at the time [16], and evidence indicates that self-reported symptoms are largely concordant with diagnosed symptomatic infection [19,20]. Loss of smell (anosmia) is a specific symptom of many COVID-19 patients, which was experienced by 55.6% of participants with three or more symptoms of COVID-19 in our study, paralleling findings among COVID-19 patients in other regions [21,22].

Given limited diagnostics in the region during the early months of the pandemic, it remains possible that government statistics also reflect an underestimate of disease burden from this early pandemic period. We note that the majority of participants who were tested for COVID-19 were employed in corn production, although we do not know if this testing was due to a disease cluster, symptoms, or surveillance. Additional information would inform a more comprehensive understanding of our testing data. Furthermore, symptoms-based assessment would clearly not depict asymptomatic infection, which may be higher than 30% of all COVID-19 infections [23,24]. Restricting cases to symptomatic persons underestimates incidence of infection, particularly among the relatively young individuals in our study who are more likely to experience asymptomatic or mild infections. However, other endemic diseases in the region, including dengue, chikungunya and leptospirosis, may present with symptoms overlapping those of COVID-19 and this is a clear limitation of our approach [25]. Likewise, symptoms-based reporting is less accurate in a setting with low prevalence of disease, reflecting further uncertainty around our disease estimates.

Although there is large statistical uncertainty, we observed higher incidence of COVID-19-associated symptoms among participants with CKD compared to those without CKD. These observations, while not determinative, may suggest that persons with CKD experience greater susceptibility to symptomatic COVID-19. Our sample size prevented us from evaluating industry as a risk factor in these adjusted COVID-19 models; however, such studies are warranted. The long-term consequences of COVID-19 infection on the progression of CKD in Central America and on kidney health among persons at risk remains an important area for our future study.

Notably, individuals with CKD experienced greater risk of food insecurity, income loss and reductions in access to medical care compared to those without CKD, although our findings do not inform the specific reasons for this association. In a region with elevated prevalence of CKD, these implications of the COVID-19 pandemic are particularly concerning. Additionally, reduced access to medical care during the pandemic poses a significant health risk to individuals with CKD, who require regular engagement with health professionals to successfully manage the disease. Loss of regular medical care is particularly devastating for individuals with more advanced disease and a critical area for intervention. Further analyses should consider specific forms of healthcare access that were lost during the pandemic and the implications for CKD patients.

We observed lower odds of COVID-19-associated symptoms among participants working in agriculture compared to those in non-agricultural industries. Evidence indicates that SARS-CoV-2 does not transmit effectively outdoors, and it is possible that our findings support this conclusion [26]. Our observations may suggest that the outdoor nature of agricultural work, even in the context of large working groups, is sufficient to reduce occupational transmission of the virus, although without molecular diagnostics, we cannot conclude as much. However, our non-agricultural workers included a mix of employment scenarios, including many employed in outdoor work (road construction) and also those presumably working indoors (drivers, security guards). Additional data would be needed to draw conclusions here.

Our study indicates significant negative effects of the early months of the pandemic on wellbeing and health of MANOS participants and their household members. Notably, more than 14% of participants reported new food insecurity during this time period and more than 5% indicated a loss of income resulting in an inability to purchase basic household necessities. These difficulties occurred despite governmental efforts to address income and food insecurity early in the pandemic. In May 2020, the El Salvadorian government initiated income and food support to households not covered by the pension system (estimated 60% of all households). These payments included $300 per household and 2.7 million food baskets to lower-income households in the spring of 2021 [27]. We assume this support reduced income and food insecurity in the country and in our study population.

The effect of new income and food insecurity could be particularly devastating on families already experiencing poverty [28,29]. In particular, food instability poses a long-term threat to child development, effects which could be amplified by conditions of poverty and pre-existing nutritional deficit among youth in the region [30,31]. Notably, the majority of those experiencing food and income insecurity were employed at the time of the interview, suggesting demonstrable economic stressors not solely associated with the participant’s work. Loss of employment for other family members, illness in the family, loss of secondary employment or diminished remittances from family members abroad may contribute to these trends.

Our study is limited to the first six months of the pandemic. It is possible that data from later months of the pandemic would reflect different experiences with COVID-19 among MANOS participants. At baseline, all MANOS participants, including those with CKD, were employed full-time. As full-time workers, the overall health of our participants may contribute to reduced susceptibility to severe COVID-19. As a result, our findings may underestimate the incidence of COVID-19 among individuals with CKDu in the region, including those who are unable to work. It is possible that the participants who we failed to reach for this study differed from those enrolled in MANOS in a way that biased our estimates, although the directionality of such a potential effect is unclear. The timeframe of this study (March through September 2020) reflected the early wave of the pandemic, but it is feasible that we underestimated COVID-19 symptoms among those who were contacted earlier in the study period when the disease was less prevalent. While there is no difference in timing of contact by CKD status, the time required to contact participants likely led to an underestimate of symptoms among those interviewed earlier in the study period. We were unable to reach all participants with CKD for this substudy; this reflects both general loss to follow up prior to the pandemic as well as potential loss specific to the pandemic or this substudy. It is possible that our estimates regarding the negative effects of the pandemic associated with CKD were attenuated as a result.

We note that our study overlapped with the final months of the sugarcane growing season and included the rainy season, during which sugarcane workers are typically employed in other industries. While we specifically queried pandemic-related employment changes as distinct from regular, seasonal changes to employment, it is possible reports from sugarcane workers incorporate both predicted and unexpected employment changes. We did not comprehensively capture transport to and from work in our study, and it is possible that associations observed are due to transportation compared to occupational exposures or other factors. It is also possible that unemployment status peaked earlier in the study months, suggesting that persons contacted earlier in the study period were more likely to report unemployment. Follow-up studies of the longer-term implications of the pandemic for employment in El Salvador would be needed to fully address these potential concerns. Finally, some COVID-19 symptoms, specifically fever, muscle ache and fatigue, may be associated with acute kidney injury (AKI) events, as there are reports of such in the literature [32,33]. It is possible that some of the COVID-19 symptoms reported among participants were associated with kidney injury and not an infectious disease. While a thorough assessment of AKI is beyond the scope of this paper, the role of AKI events in contributing to COVID-19 is worthy of additional study. Additional clinical and epidemiological data regarding COVID-19 among persons with CKD is necessary to draw conclusions as to the vulnerability of this patient population to COVID-19.

## Conclusion

Findings from our study identified significant economic disruption and morbidity within this working-age cohort in El Salvador during the first six months of the pandemic. There is scant information on incidence or community health in Central America during the pandemic, and in many regions the COVID-19 pandemic is overlaid with an ongoing epidemic of CKD in the region. The intersections of these crises warrant immediate intervention. Continued attention to Central American communities is necessary to attenuate the effects of the COVID-19 pandemic.

## Data Accessibility Statement

The datasets generated during and/or analyzed during the current study are available from the corresponding author on reasonable request.
